# Circulating miRNAs as Novel Diagnostic Biomarkers in Nonalcoholic Fatty Liver Disease: A Systematic Review and Meta-Analysis

**DOI:** 10.1155/2019/2096161

**Published:** 2019-08-20

**Authors:** Changzhou Cai, Yiming Lin, Chaohui Yu

**Affiliations:** Department of Gastroenterology, The First Affiliated Hospital of Zhejiang University School of Medicine, Hangzhou 310003, China

## Abstract

**Background and Aims:**

Recent studies have indicated that circulating miRNAs could serve as accurate biomarkers for diagnosing nonalcoholic fatty liver disease (NAFLD). We aimed to assess the evidence on the probability of circulating miRNAs as new diagnostic biomarkers in patients with NAFLD.

**Methods:**

We comprehensively retrieved relevant English literature from the databases of PubMed, Embase, and the Cochrane Library from 2000 to 1 January 2019. The diagnostic accuracy of circulating miRNAs as markers for NAFLD was analyzed. Moreover, we evaluated the methodological quality of the included article. STATA was applied to perform statistical analyses.

**Results:**

In this meta-analysis, 17 studies that enrolled 1408 patients of NAFLD and 926 healthy people from 6 articles were analyzed. We constructed a summary receiver-operating characteristic (SROC) curve of all circulating miRNAs, and the area under the curve (AUC) was 0.83, with the pooled sensitivity (SEN) 0.70 and the pooled specificity (SPE) 0.82 in distinguishing patients with NAFLD from healthy controls. Among them, miR-122 showed high diagnostic accuracy, with the diagnostic index of pooled SEN, SPE, and AUC being 0.88, 0.66, and 0.86, respectively. We then performed subgroup analyses based on the mode of miRNA regulation, countries, miRNA profiling, sample size, and male proportion. We then did a regression analysis and found the cause of heterogeneity might be miRNA profiling. Finally, publication bias was not found, and Fagan's nomogram showed valuable clinical utility.

**Conclusion:**

Circulating miRNAs, especially miR-122, might be promising diagnostic biomarkers for NAFLD with high-accuracy, and more large-sample studies are required to support the above findings in the future.

## 1. Introduction

Currently, nonalcoholic fatty liver disease (NAFLD) is gradually becoming one of the most common chronic liver diseases in the world [1]. It is predicted to become the leading cause of liver transplantation, end-stage liver disease by 2025, and histological ranges from simple steatosis (SS) to steatohepatitis and fibrosis [2, 3]. Up to now, the diagnosis and staging of NAFLD still depended on the gold index of liver biopsy, which is an invasive technique with the risk of complications such as bleeding. It also shows up intrinsic sampling variability because of the limitations of the biopsy area compared to the entire liver [4]. Therefore, it is urgent to find out high-accuracy noninvasive biomarkers for primary diagnosis of NAFLD clinically.

MicroRNAs (miRNAs) are small (18 to 24 nucleotides long), endogenous, noncoding RNA molecules. MicroRNAs can act at posttranscriptional levels, targeting downstream genes and affecting their protein synthesis [5]. Besides, because protected by RNase, miRNAs are stable in clinical blood samples and circulating miRNAs can indirectly reflect the status of the original tissue. Therefore, researchers have recently proposed miRNAs detected in circulation as noninvasive probable diagnostic biomarkers for distinguishing patients from healthy ones [6].

A series of theoretical studies have reported that changes in circulating miRNA expression are related to the occurrence and development of NAFLD and many studies used serum/plasma miRNAs as noninvasive biomarkers for diagnosing NAFLD [7–10]. However, there is still no clear consensus on the possibility of circulating miRNAs as diagnostic indicators for NAFLD. In this study, we comprehensively searched the literature to elucidate the accuracy of miRNAs in the diagnosis of NAFLD patients and offer new referential information for early diagnosis of NAFLD.

## 2. Materials and Methods

### 2.1. Literature Retrieval

We performed this study based on the preferred reporting items for systematic reviews and meta-analysis (PRISMA) ([Supplementary-material supplementary-material-1]) guidelines. We searched relevant English articles from PubMed, Embase, and the Cochrane Library, with the final literature published before 1 January 2019. The retrieval strategy was used as follows: (“NAFLD” OR “Non-alcoholic Fatty Liver Disease” OR “NASH” OR “Non-alcoholic Steatohepatitis”) AND (“microRNAs” OR “miRNA” OR “microRNA” OR “miR” OR “hsa-miR”). Moreover, the two reviewers manually retrieved and browsed the appropriate studies that may exist, and eventually, we did not include additional literature in the study.

### 2.2. Literature Inclusion and Exclusion Criteria

We included studies in the following conditions: (1) studies regarding microRNAs comparing NAFLD patients with healthy controls; (2) patients and controls in all studies were not limited by race or age, and liver biopsy was the diagnostic gold index for all NAFLD patients; (3) the information of sample size, sensitivity (SEN), specificity (SPE), or enough data was given to build a diagnostic 2 *∗* 2 contingency table. We excluded studies in the following conditions: (1) the reports were cell or animal studies, reviews, meetings, and letters; (2) the pathological diagnosis standard was not liver biopsy; (3) the studies were duplicated.

### 2.3. Procedure

Two reviewers selected studies by reviewing titles and abstracts as well as full text and extracted data, independently. Discrepancies were resolved by referral to a third reviewer. The retrieved data included the following: (1) necessary information including publication year, the first author, ethnicity, target microRNAs, regulation mode of miRNAs, sample size, and male ratio; (2) diagnostic parameters of the microRNAs, including SEN and SPE.

We scored the methodological quality of the included studies in our research by following the revised tool for the quality assessment of diagnostic accuracy studies (QUADAS) [11, 12] checklist in Review Manager Version 5.3. Patient selection, index test, reference standard, and ﬂow and timing were the four assessment projects. The questions contained in each section can be judged by the “yes,” “no,” or “unclear” arguments. Answering “no” or “not clear” will be considered the high risk of bias, whereas the answer of “yes” will be viewed as a relatively low risk of bias. Three aspects concerning reference, case selection, and inspection were used to evaluate the applicability of the articles.

### 2.4. Statistical Analysis

After extracting the data of SEN, SPE, case numbers, and control numbers, we built the diagnostic 2 *∗* 2 contingency table and calculated the numbers of true positives, false positives, false negatives, and true negatives in patients from each study. We assessed the heterogeneity among studies by *I* ^2^ statistic. If the *I* ^2^ value was more than 50%, we would choose the random-effects model. We then performed subgroup analyses based on the mode of miRNA regulation, countries, miRNA profiling, sample size, and male proportion. Regression analysis was conducted to explore the causes of heterogeneity. By further summarizing the data from SEN, SEN, positive likelihood (PLR), negative likelihood (NLR), and the diagnostic odds ratio (DOR), we assessed the diagnostic efficiency of miRNAs. Besides, we plotted the summary receiver-operating characteristics (SROC) curve through STATA software. And the area under the SROC curve (AUC) of overall and subgroup analyses were calculated. We then constructed Deeks' funnel plots used for detecting publication bias, and *P* < 0.10 was considered having publication bias. Finally, we erected Fagan's nomogram plots used for assessing clinical utility. All of these were performed by STATA version 12.

## 3. Results

### 3.1. Study Selection and Literature Characteristics

**Figure 1:**
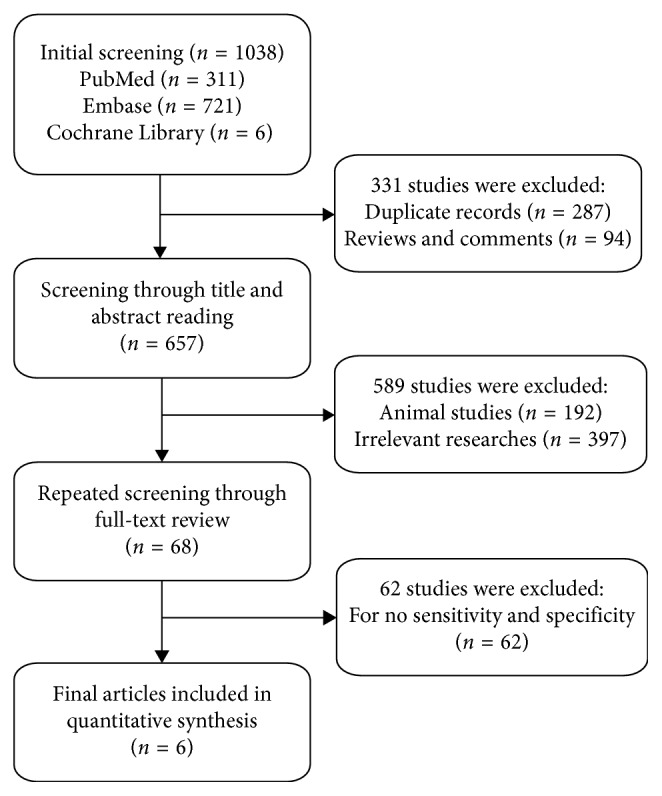
Flow chart of this systematic review and meta-analysis to identify inclusion studies.

**Figure 2:**
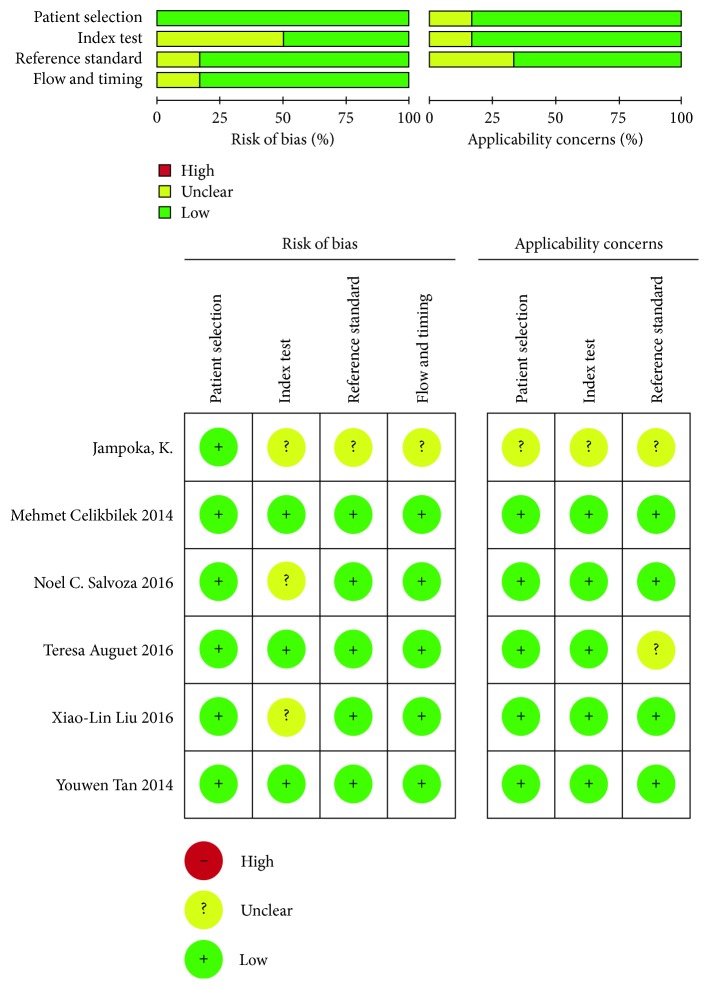
Overall methodology quality assessment of included articles using the QUADAS criteria.

Following the literature electronic searching strategy, we retrieved 1038 articles, of which 721 were from Embase, 311 were from Pubmed, and six were from Cochrane. We then removed 287 duplicates, 94 reviews, 192 animal research, 397 irrelevant studies, and 62 articles without available diagnostic information (Figure 1). Eventually, we adopted 17 studies from six items [13–18] in our research, 12 studies from five articles were performed to analyze the diagnostic accuracy of circulating microRNAs for NAFLD (including NAFL and NASH), and five studies from two articles were conducted to analyze the diagnostic efficiency of circulating microRNAs for NASH. The data of the 17 studies were listed in Table 1. In all, this meta-analysis included 1408 patients with NAFLD and 926 healthy controls (HCs). Among these 17 studies, there were 16 studies on single microRNAs and the other on multi-miRNAs. Besides, the most studied miRNAs included miR-122, miR-34a, and miR-99a. And quantitative real-time reverse transcription-PCR (qRT-PCR) was performed to identify the miRNA expression level from serum specimens in all studies. We displayed the methodological quality of each included articles assessed by QUADAS in a bar chart (Figure 2).

### 3.2. Diagnostic Value of Circulating MicroRNAs in NAFLD Patients

**Figure 3:**
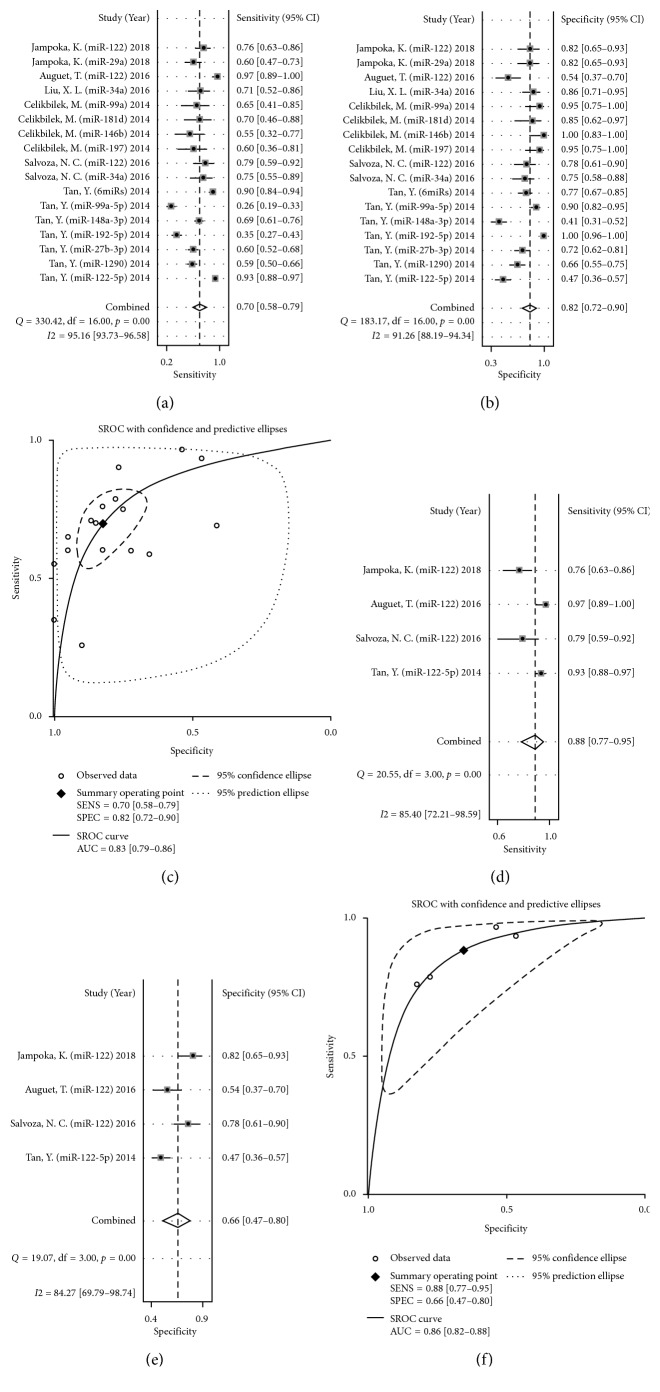
Forest plots of sensitivity (a), specificity (b), and area under the curve (c) of circulating miRNAs for diagnosing nonalcoholic fatty liver disease among 17 studies. Forest plots of sensitivity (d), specificity (e), and area under the curve (f) of microRNA-122 for diagnosing nonalcoholic fatty liver disease among four studies.

**Figure 4:**
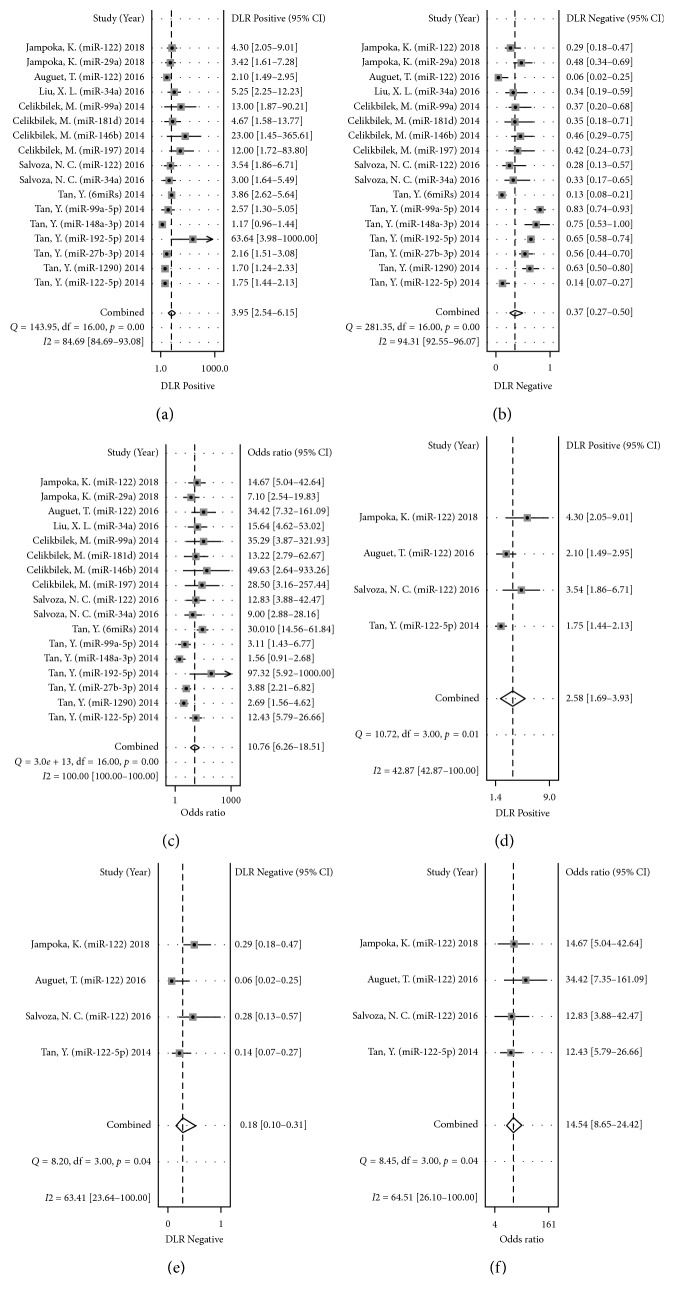
Forest plots of positive likelihood ratio (a), negative likelihood ratio (b), and diagnostic odds ratio (c) of circulating miRNAs for diagnosing nonalcoholic fatty liver disease among 17 studies. Forest plots of positive likelihood ratio (d), negative likelihood ratio (e), and diagnostic odds ratio (f) of microRNA-122 for diagnosing nonalcoholic fatty liver disease among four studies.

In our meta-analysis, the data of SEN and SPE from 17 miRNAs studies were summarized and displayed in the form of forest plots. Significant heterogeneity existed among the studies from the data of sensitivity and specificity (*I* ^2^ = 95.16% and *I* ^2^ = 91.26%, respectively) (Figure 3), and hence, we chose the random-effects model in our analyses. The pooled data were displayed: SEN, 0.70 (95% CI: 0.58–0.79) (Figure 3(a)); SPE, 0.82 (95% CI: 0.72–0.90) (Figure 3(b)); PLR, 3.95 (95% CI: 2.54–6.15) (Figure 4(a)); NLR, 0.37 (95% CI: 0.27–0.50) (Figure 4(b)); DOR, 10.76 (95% CI: 6.26–18.51) (Figure 4(c)); and AUC, 0.83 (95% CI: 0.79–0.86) (Figure 3(c)) (Table 1).

Besides, we investigated the value of circulating miRNAs in the diagnosis of NAFLD (including NAFL and NASH) and NASH alone, separately (Table 2). The pooled data for diagnosing NAFLD were as follows: SEN, 0.72 (95% CI: 0.57–0.84); SPE, 0.76 (95% CI: 0.62–0.86); PLR, 3.05 (95% CI: 2.01–4.62); NLR, 0.36 (95% CI: 0.24–0.55); DOR, 8.40 (95% CI: 4.63–15.24); and AUC, 0.81 (95% CI: 0.77–0.84). And the pooled data for diagnosing NASH were as follows: SEN, 0.65 (95% CI: 0.55–0.73); SPE, 0.92 (95% CI: 0.83–0.97); PLR, 8.27 (95% CI: 3.74–18.31); NLR, 0.38 (95% CI: 0.29–0.50); DOR, 21.62 (95% CI: 8.71–53.66); AUC, 0.80 (95% CI: 0.76–0.83). These results indicated that circulating miRNAs have similar and high diagnostic efficiency in the diagnosis of NAFLD (not distinguishing between NAFL and NASH) and NASH.

Among them, miR-122 was reported in 4 studies, and we pooled the data of SEN, SPE, PLR, NLR, and DOR, which were 0.88 (95% CI: 0.77–0.95) (Figure 3(d)), 0.66 (95% CI: 0.47–0.80) (Figure 3(e)), 2.58 (95% CI: 1.69–3.93) (Figure 4(d)), 0.18 (95% CI: 0.10–0.30) (Figure 4(e)), and 14.54 (95% CI: 8.65–24.42) (Figure 4(f)), respectively. Then, we constructed the SROC, and the AUC was 0.86 (95% CI: 0.82–0.88) (Figure 3(f)). These results revealed high accuracy of circulating miRNAs in diagnosing patients with NAFLD, especially miR-122.

### 3.3. Subgroup Analyses and Regression Analysis

To refine the results, we performed subgroup analyses based on the mode of miRNA regulation, countries, miRNA profiling, sample size, and male proportion. Table 2 shows the pooled data of SEN, SPE, PLR, NLR, DOR, and AUC for each subgroup analysis, in which we found that upregulated miRNAs exhibited higher diagnostic accuracy than downregulated miRNAs, with SEN (0.73 vs. 0.61), SPE (0.77 vs. 0.91), DOR (8.99 vs. 17.04), and AUC (0.82 vs. 0.65). Results from non-Chinese studies also exhibited higher diagnostic accuracy than Chinese studies, with SEN (0.73 vs. 0.66), SPE (0.83 vs. 0.79), DOR (13.46 vs. 7.58), and AUC (0.86 vs. 0.79). Besides, the assay using the miRNA panel exhibited higher diagnostic accuracy than assays using single miRNA, with SEN (0.90 vs. 0.68), SPE (0.76 vs. 0.83), DOR (9.13 vs. 9.96), and AUC (0.89 vs. 0.82). Apart from that, the studies with male proportion less than 50 percent were slightly better than the studies with male proportion more than 50 percent in diagnosing NAFLD, with SEN (0.77 vs. 0.69), SPE(0.81 vs. 0.78), DOR (25.90 vs. 7.88), and AUC (0.90 vs. 0.80). Sample size did not influence the diagnosis (Table 2).

To find out possible reasons for heterogeneity, we performed metaregression and used logOR as the dependent variable. And we considered regulation mode, miRNA profiling, country, and sample size as covariates. The result of *I*-squared_res value was 64.66%, indicating the heterogeneity existed between studies, and the reason for heterogeneity might be related to the miRNA profiling (*P*=0.040) (Table 3) but was unrelated to the mode of regulation, country, and sample size. Due to lack of some data, the male ratio was not involved in regression analysis (Table 1). When excluded the study of miRNAs panel, heterogeneity *I*-squared_res decreased from 80.9% to 75.9%. Besides, the pooled data of SEN, SPE, PLR, NLR, DOR, and AUC were 0.68 (95% CI: 0.56–0.78), 0.83 (95% CI: 0.71–0.90), 3.90 (95% CI: 2.42–6.28), 0.39 (95% CI: 0.29–0.52), 9.96 (95% CI: 5.67–17.52), and 0.82 (95% CI: 0.78–0.85), respectively ([Supplementary-material supplementary-material-1]). And Fagan's nomogram is presented in [Supplementary-material supplementary-material-1].

### 3.4. Publication Bias

Potential publication bias was assessed by conducting Deeks' funnel plots. The pooled Deeks' test results of all studies were *t* = 1.14 and *P*=0.272 ([Supplementary-material supplementary-material-1]), and the consequences of miR-122 were *t* = 0.58 and *P*=0.623 ([Supplementary-material supplementary-material-1]), which demonstrated no significant publication bias in this analysis.

### 3.5. Clinical Utility Analyses

**Figure 5:**
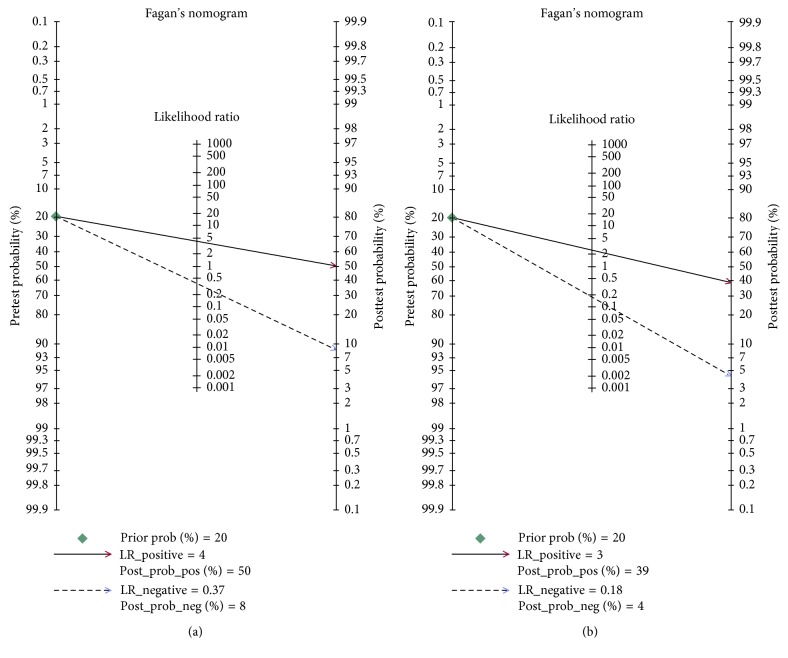
Fagan's nomogram of circulating miRNAs (a) and microRNA-122 (b) for diagnosing nonalcoholic fatty liver disease.

The clinical utility of circulating miRNAs was evaluated by conducting Fagan's nomogram. As is shown in Figure 5(a), the results indicated that if we set the pretest probability at 20%, the PLR was four accompanied by the posttest chance of 50% and the NLR was 0.37 accompanied by the posttest probability would decrease to 8%. As for miR-122 (Figure 5(b)), if we set the pretest probability at 20%, the PLR was three accompanied by the posttest chance of 39% and NLR was 0.18 accompanied by the posttest probability of 4%.

## 4. Discussion

Nowadays, NAFLD is gradually becoming one of the most common chronic liver diseases in the world and the leading cause of end-stage liver disease, liver transplantation, and HCC [19]. Up to now, liver biopsy is still the gold index for diagnosing and staging of NAFLD but hard to implement because of its invasiveness and various complications. Besides, imaging techniques including ultrasound and computed tomography scans were the most common methods for assessing hepatic steatosis but have limitations in the diagnosis of mild hepatic steatosis [20]. Thus, it is urgent to develop reliable noninvasive biomarkers for primary diagnosis of NAFLD clinically with a new perspective. Recently, some new noninvasive diagnostic markers have emerged, including noncoding RNAs, circulating protein, and gene mutations [21]. Some studies indicated that the potential values of miRNAs in the diagnosis of NAFLD are similar or even superior when compared with other biomarkers, CK18, for instance [22]. And the diagnostic accuracy in NAFLD could be improved by combining multiple circulating miRNAs [22]. Therefore, the researcher gradually proposed the role of circulating miRNAs in diagnosing patients with NALFD. This article is the first innovative meta-analysis of studying circulating miRNAs acting as diagnostic biomarkers for NAFLD.

We retrieved relevant articles about studying the association between circulating miRNAs and NAFLD. Twelve miRNAs concerning the diagnostic value on NAFLD were included in this study. The efficiency and suitability of circulating miRNAs acting as diagnostic biomarkers for NAFLD were evaluated by pooling the data of SEN, SPE, DOR, and AUC. As for the overall results of circulating miRNA assays in patients with NAFLD, the pooled diagnostic parameters of SEN, SPE, DOR, and AUC values were 0.70 (95% CI: 0.58–0.79), 0.82 (95% CI: 0.72–0.90), 10.76 (95% CI: 6.26–18.51), and 0.83 (95% CI: 0.79–0.86), respectively. Among them, four articles [13, 14, 17, 18] including 12 studies focused on the diagnostic accuracy of circulating microRNAs for NAFLD (including NAFL and NASH), and two articles [15, 16] including 5 studies focused on the diagnostic accuracy of circulating microRNAs for NASH. And pooled results indicated that circulating miRNAs have similar and high diagnostic efficiency in the diagnosis of NAFLD (not distinguishing between NAFL and NASH) and NASH. The above results manifested the potential applicability of circulating miRNAs in diagnosing NAFLD. In this review and analysis, there were 7 upregulated miRNAs including miR-122, miR-99a-5p, miR-34a, miR-1290, miR-27b-3p, miR-192-5p, and miR-148a-3p and 5 downregulated miRNAs, including miR-197, miR-146b, miR-181d, miR-99a, and miR-29a. Our results also confirmed the high diagnostic accuracy of miR-122 defined by high SEN and SPE, making it a potential diagnostic biomarker for NAFLD (sensitivity: 0.88; specificity: 0.66). Many studies found that miR-122 was widely involved in various physiological processes of the liver and miR-122 had been receiving much attention. A cross-sectional study of 443 subjects enrolled in a Japanese health check found five elevated serum levels of miRNA in NAFLD patients, including miR-122, miR-21, miR-34a, miR-145, and miR-451, in which the circulating miR-122 expression levels associated with the progression of NAFLD [23]. Besides, circulating miR-122 expression levels were found upregulated in high-fat-diet (HFD) feeding mice and changed in the early-stage of NAFLD, therefore indicating that miR-122 could act as a novel primary biomarker in the diagnosis of NAFLD [24]. In another animal study, circulating miR-122 expression levels were found to be increased by 40-fold in the mouse model fed with methionine-choline-deficient (MCD) diet in the early time [6]. Besides, in a study including total 67 NAFLD subjects, when compared with simple steatosis patients, circulating miR-122 expression levels were higher in NASH patients and might associate with the degree of inflammation and fibrosis grading [25]. Another study showed the same results that serum miR-122 expression levels were increased in NASH subjects when compared with simple steatosis patients [26].

Similarly, miR-122 was observed exhibiting better diagnostic performance in NASH and liver fibrosis in NAFLD patients than cytokeratin- (CK-) 18 and AST or ALT [7]. However, many other liver diseases also found increased circulating miR-122 expression levels, including chronic hepatitis B and chronic hepatitis C, alcoholic liver disease, and drug-induced liver injury. Thus, the specificity of circulating miR-122 in diagnosing NAFLD may be low [27–30].

Apart from that, we conducted subgroup analyses according to the mode of miRNA regulation, country, miRNA profiling, sample size, and the proportion of males. We found that upregulated miRNAs assays exhibited higher diagnostic accuracy than downregulated miRNAs assays (AUC, 0.82 vs. 0.65). Non-Chinese tests showed higher diagnostic accuracy than Chinese criteria (AUC, 0.86 vs. 0.79). The analysis using the miRNA panel exhibited higher diagnostic accuracy than single miRNA (AUC, 0.89 vs. 0.82). And the studies with the proportion of males less than 50 percent were slightly better than the studies with the percentage of males more than 50 percent in the diagnosis of NAFLD (AUC, 0.90 vs. 0.80). Given that, we may conclude that greater diagnostic values, performing higher AUC, can be achieved in the non-Chinese female population using the upregulated miRNA panel. However, we should note the considerable heterogeneity in the included studies. Subsequently, we performed metaregression according to the variables, including regulation mode, miRNA profiling, country, and total sample size. The results indicated that the reason for heterogeneity might relate to miRNA profiling, but not the regulation mode, country type, and sample size. When we excluded the miRNA panel study, the heterogeneity chi-squared decreased from 82.91 to 61.08 and *I*-squared decreased from 80.7% to 75.4%, partially explaining the source of heterogeneity. On account of insufficient data, we could not further assess other potential variables that may affect the heterogeneity between studies, such as age, male ratio, and disease severity.

In spite of our efforts to analyze comprehensively and accurately, there are still some defects in this study: (1) we may have ignored some relevant literature or part of the data; (2) several other variables such as age, male ratio, and disease severity could not be further assessed due to lacking sufficient information; (3) different values of cutoff and circulating miRNAs may cause heterogeneity and different results.

In conclusion, this systematic review and meta-analysis showed the promising biomarkers of circulating miRNAs in distinguishing patients with NAFLD from healthy controls with high diagnostic indicators. The most studied circulating miRNA, circulating miR-122, may be a highly accurate diagnostic tool for NAFLD. More rigorous randomized controlled studies are needed to validate the above conclusions.

## Figures and Tables

**Table 1:** Characteristics of the included studies.

First author	Year	Country	MicroRNAs	Regulation mode	Disease	Sample size		Male *N* (%)	Diagnostic power	
						Case	Control		Sen (%)	Spe (%)
Y. Tan	2014	China	miR-122-5p	Upregulated	NAFLD	152	90	195 (80.5)	93.4	46.7
Y. Tan	2014	China	miR-1290	Upregulated	NAFLD	152	90	195 (80.5)	58.6	65.6
Y. Tan	2014	China	miR-27b-3p	Upregulated	NAFLD	152	90	195 (80.5)	59.9	72.7
Y. Tan	2014	China	miR-192-5p	Upregulated	NAFLD	152	90	195 (80.5)	34.9	93.3
Y. Tan	2014	China	miR-148a-3p	Upregulated	NAFLD	152	90	195 (80.5)	69.1	41.1
Y. Tan	2014	China	miR-99a-5p	Upregulated	NAFLD	152	90	195 (80.5)	25.7	90
Y. Tan	2014	China	6miRs panel	Upregulated	NAFLD	152	90	195 (80.5)	90.3	76.2
N. C. Salvoza	2016	America	miR-34a	Upregulated	NAFLD	28	36	35 (54.7)	75	75
N. C. Salvoza	2016	America	miR-122	Upregulated	NAFLD	28	36	35 (54.7)	78.6	77.8
M. Celikbilek	2014	Turkey	miR-197	Downregulated	NASH	20	20	18 (45.0)	60	95
M. Celikbilek	2014	Turkey	miR-146b	Downregulated	NASH	20	20	18 (45.0)	55	100
M. Celikbilek	2014	Turkey	miR-181d	Downregulated	NASH	20	20	18 (45.0)	70	85
M. Celikbilek	2014	Turkey	miR-99a	Downregulated	NASH	20	20	18 (45.0)	65	95
X. L. Liu	2016	China	miR-34a	Upregulated	NASH	31	37	40 (58.8)	70.4	87.5
T. Auguet	2016	Spain	miR122	Upregulated	NAFLD	61	39	0 (0.0)	96.6	54, 7
K. Jampoka	2018	Thailand	miR-29a	Downregulated	NAFLD	58	34	—	60.37	82.35
K. Jampoka	2018	Thailand	miR-122	Upregulated	NAFLD	58	34	—	75	82.35

Sen: sensitivity; Spe: specificity.

**Table 2:** Summary estimates of diagnostic power and their 95% confidence intervals.

Subgroup		Sensitivity (95% CI)	Specificity (95% CI)	Positive LR (95% CI)	Negative LR (95% CI)	DOR (95% CI)	AUC
*Regulation mode*							
Upregulated	12	0.73 [0.58–0.84]	0.77 [0.63–0.87]	3.15 [2.04–4.85]	0.35 [0.23–0.54]	8.99 [4.88–16.75]	0.82 [0.78–0.85]
Downregulated	5	0.61 [0.53–0.69]	0.91 [0.81–0.96]	7, 18 [3.06–16.71]	0.42 [0.34–0.53]	17.04 [6.49–44.75]	0.65 [0.61–0.69]

*Country*							
Chinese	8	0.66 [0.47–0.82]	0.79 [0.58–0.91]	3.21 [1.62–6.36]	0.42 [0.27–0.67]	7.58 [3.24–17.78]	0.79 [0.75–0.82]
Non-Chinese	9	0.73 [0.61–0.82]	0.83 [0.74–0.90]	4.36 [2.98–6.36]	0.32 [0.23–0.46]	13.46 [8.48–21.26]	0.86 [0.82–0.89]

*miRNA profiling*							
miRNA panel	1	0.90	0.76	3.86	0.42	9.13	0.89
Single miRNA	16	0.68 [0.56–0.78]	0.83 [0.71–0.90]	3.90 [2.42–6.28]	0.39 [0.29–0.52]	9.96 [5.67–17.52]	0.82 [0.78–0.85]

*Sample size*							
≥100	9	0.72 [0.53–0.85]	0.77 [0.59–0.88]	3.05 [1.84–5.06]	0.37 [0.23–0.60]	8.19 [4.06–16.54]	0.81 [0.77–0.84]
<100	8	0.69 [0.60–0.76]	0.89 [0.79–0.94]	5.96 [3.20–11.12]	0.36 [0.28–0.46]	16.67 [8.27–33.98]	0.81 [0.78–0.84]

*Male proportion*							
>50%	10	0.69 [0.52–0.81]	0.78 [0.62–0.89]	3.16 [1.87–5.35]	0.40 [0.27–0.60]	7.88 [3.97–15.63]	0.80 [0.76–0.83]
≤50%	5	0.77 [0.69–0.84]	0.81 [0.72–0.87]	5.92 [1.96–17.85]	0.35 [0.22–0.54]	25.90 [10.99–61.06]	0.90 [0.88–0.92]

*Disease*							
NAFLD	12	0.72 [0.57–0.84]	0.76 [0.62–0.86]	3.05 [2.01–4.62]	0.36 [0.24–0.55]	8.40 [4.63–15.24]	0.81 [0.77–0.84]
NASH	5	0.65 [0.55–0.73]	0.92 [0.83–0.97]	8.27 [3.74–18.31]	0.38 [0.29–0.50]	21.62 [8.71–53.66]	0.80 [0.76–0.83]
Total	17	0.70 [0.58–0.79]	0.82 [0.72–0.90]	3.95 [2.54–6.15]	0.37 [0.27–0.50]	10.76 [6.26–18.51]	0.83 [0.79–0.86]

LR: likelihood ratio; DOR: diagnostic odds ratio; AUC: area under the curve; CI: confidence interval.

**Table 3:** Metaregression analysis in the binary classification of variable data using the odds ratio (OR).

LogOR	Exp(*b*)	Std. error	*t*	*P* > |*t*|	(95% conf. interval)	
Regulation mode	0.0059773	0.6970161	0.01	0.993	−1.51269	1.524645
Country	−0.9125667	0.6038116	−1.51	0.157	−2.228159	0.4030257
miRNA profiling	1.916451	0.8301294	2.31	0.040	0.1077546	3.725148
Sample size	−0.55473	.5707856	−0.97	0.350	−1.798365	0.6889049

We used LogOR as response variables and used the regulation mode, miRNA profiling, country, and sample size as covariates. Estimate of between-study variance tau^2^ = 0.4247. Residual variation due to heterogeneity: *I*-squared_res = 64.66%. Proportion of between-study variance explained: Adj. *R*-squared = 48.15%. Joint test for all covariates with Knapp–Hartung modification: Prob > *F* = 0.0964.

## Data Availability

The data used to support the findings of this study are available from the corresponding author upon request.

## Abbreviations

NAFLD: Nonalcoholic fatty liver disease
miRNAs: MicroRNAs
SEN: Sensitivity
SPE: Specificity
PLR: Positive likelihood ratio
NLR: Negative likelihood ratio
SROC: Summary receiver-operating characteristics
AUC: Area under the curve of ROC
DOR: Diagnostic odds ratio
OR: Odds ratio
qRT-PCR: Quantitative reverse transcription polymerase chain reaction
ALT: Alanine aminotransferase
AST: Aspartate aminotransferase
HCC: Hepatocellular carcinoma.

## Supplementary Materials

Supplementary Materials Table S1: preferred reporting items for systematic reviews and meta-analysis (PRISMA) guidelines. Figure S1: forest plots of sensitivity, specificity, positive likelihood ratio (PLR), negative likelihood ratio (NLR), diagnostic odds ratio (DOR), area under the curve (AUC), funnel plot, and Fagan's nomogram of circulating miRNAs for diagnosing nonalcoholic fatty liver disease among 16 studies after excluding the miRNA panel. (A) Sensitivity; (B) specificity; (C) PLR; (D) NLR; (E) DOR; (F) AUC; (G) funnel plot; and (H) Fagan's nomogram. PLR: positive likelihood ratio; NLR: negative likelihood ratio; DOR: diagnostic odds ratio; AUC: area under the curve. Figure S2: publication bias assessed by Deeks' test. (A) Publication funnel plots of overall circulating miRNAs for diagnosing non-alcoholic fatty liver disease; (B) publication funnel plots of overall circulating mi-122 for diagnosing non-alcoholic fatty liver disease
